# The Bloodless Method of Performing Surgical Operations

**Published:** 1881-03

**Authors:** 


### EXCERPTA.

THE BLOODLESS METHOD OF PERFORMING SURGICAL OPERATIONS.—Prof. Esmarch, of Kiel, again alluded to this subject at the Congress of German Surgeons at Berlin. Reprint from *Langenbeck's Archiv*, (vol. xxv). He remarked that seven years had now elapsed since he had first advocated the use of the bloodless method for surgical procedures. Since then most surgeons had given his method a trial. Many eminent and experienced physicians had, however, abandoned it on account of the secondary hemorrhage following the removal of the constricting bandage. This was to be regretted, for none could deny the great advantages to be gained by artificial ischemia. Esmarch attributed to a defective *technique* the disadvantages claimed to be associated with his method. Nor did he think that the modifications variously suggested were calculated to obviate failures from this source. He had himself been occupied with further improvements, and had been so far successful that he performed most of his recent operations on the extremities with absolutely no loss of blood. The present mode of procedure in the three principal varieties of operations on the limbs—*i. e.*, amputations, excisions and sequestrotomies—was then described. In amputations all the visible vessels were carefully tied, but the constricting tube was at once removed. The edges of the wound were then united by deep catgut sutures, after which a short drainage-tube, capable of absorption, was fixed at the most dependent portion of the wound; finally, a compressing retentive dressing (*comprimirender Dauev verband*) was applied, and then the constricting tube was removed, the limb being maintained in a vertical position. The patient having been placed in bed, his stump was kept in the vertical position for half an hour longer, after which time it was placed horizontally. He had performed twelve amputations in this way, and in no instance was consecutive hemorrhage observed. The dressings remained in situ for two weeks. At the end of this time they were removed, and a narrow streak of blood was all that was found to correspond to the cicatrix. In exsections he had formerly applied the dressing before removal of the constricting tube, but he had afterward abandoned this plan, owing to the occurrence of secondary hemorrhages in consequence of incomplete compression. Recently, however, he had again adopted this former method, because now he had improved the method of dressing. At present he secured the visible vessels after the operation, and proceeded in essentially the same manner as in amputations, the limb being secured to a suitable splint in the vertical position, and so held for about half an hour, after which time the patient was allowed to place it in a more comfortable position. In this way, since 1878, he had performed fifty-six exsections, and no case had secondary hemorrhage taken place. In thirty-three cases the dressing were left undisturbed for from three to four weeks. As regarded sequestrotomies, he had abandoned his former method of plugging the osseous cavities with carbolized cotton, etc. At present his method was as follows: The bone cavity was thoroughly cleansed with solutions of carbolic acid and chloride of zinc; the edges of the wound were then united by deep catgut sutures, drainage tubes capable of absorption inserted, and the constricting tube removed after completion of the retentive dressing. He had employed this method successfully in twelve cases, and in no instance had secondary hemorhage necessitated an early renewal of the first dressing. Similar dressings were applied in a precisely similar manner in such operations as the extirpation of tumors of the limbs, nerve stretchings, etc. The results in all cases were very satisfactory, which he attributed to the absence of hemorrhage, either primary or secondary.—*New York Medical Record.*

